# John Johnstone

**Published:** 1837-04

**Authors:** 


JOHN JOHNSTONE, M.D. F.R.S.

Lately, at Birmingham, Dr. John Johnstone, in his sixty-ninth year, one of the most distinguished members of a family long eminent for moral worth and scientific acquirements. Dr. Johnstone had practised as a physician in Birmingham upwards of forty years, and among the members of his profession his name must be placed in the first rank. With deep professional learning he possessed an acuteness of intellect, an insight into character, a decision of mind, and a kindness of manner, eminently valuable in every relation of life, but more peculiarly important in that of a physician. His skill was uniformly acknowledged by his fellow-citizens, and, indeed, throughout the extensive district in which he practised. But it was not as a physician only that Dr. John Johnstone was distinguished. The confidential friend and biographer of Dr. Parr was himself a scholar of no ordinary acquirements, and his biographical memoirs of that celebrated man display sound judgment, refined taste, and classical learning. The elegance, as well as the depth of his scholarship, made him the delight as well as the ornament of society, and procured for him the friendship and esteem of many of the most learned and illustrious persons in the empire. He was a zealous and firm supporter of the charitable institutions of Birmingham, and ever ready to afford the most kind and liberal encouragement to merit and talent, especially among the junior members of his profession. His services for a long period as one of the physicians to the General Hospital, were always highly appreciated, and the pupils of that institution will long remember and deplore his loss. He was a warm, sincere, and generous friend, and exemplary in all the relations of private life. Dr. John Johnstone was educated at Merton College, Oxford, was a magistrate for the counties of Warwick and Worcester, a Fellow of the Royal College of Physicians, and of the Royal Society.

